# Intestinal microbial ecology and hematological parameters of broiler fed cassava waste pulp fermented with *Acremonium charticola*

**DOI:** 10.14202/vetworld.2017.324-330

**Published:** 2017-03-18

**Authors:** Sugiharto Sugiharto, Turrini Yudiarti, Isroli Isroli, Endang Widiastuti, Fatan Dwi Putra

**Affiliations:** Department of Animal Science, Faculty of Animal and Agricultural Sciences, Diponegoro University, Semarang, Central Java, Indonesia

**Keywords:** *Acremonium charticola*, broiler, fermented cassava pulp, hematological profile, intestinal microbial ecosystem, probiotics

## Abstract

**Aim:**

The aim of this study was to evaluate the effect of feeding *Acremonium charticola*-fermented cassava pulp (AC-FCP) on the intestinal microbial ecology and hematological indices of broiler chickens.

**Materials and Methods:**

A total of 240 male Lohman day-old-chicks were randomly allotted to one of the four experimental diets including control diet, control diet + antimicrobials (neomycin; 300 mg/kg diet), diet containing AC-FCP (16 g/100 g diet) or diet containing AC-FCP + antimicrobials. At day 28, the birds from each pen were blood sampled, sacrificed and immediately the internal organs were removed and weighed. Digesta were obtained from the ileum and cecum.

**Results:**

Birds fed AC-FCP had lower (p<0.05) coliform bacteria count in the ileal digesta than birds fed control diet or control diet + antimicrobials. Butiric acid was higher (p<0.05) in the cecal content of birds fed AC-FCP than in other birds. Propionic acid was also higher in AC-FCP fed birds than in other birds although statistically not significant. The percentages of lymphocytes and heterophils were higher (p<0.05) and tended (p=0.07) to be lower, respectively, in broilers fed control diet than in other birds. The birds provided control diet had lower (p<0.05) heterophils to lymphocytes ratio compared to those receiving AC-FCP or AC-FCP + antimicrobials. Serum total protein and globulin were higher (p<0.01) in birds fed control diet or control diet + antimicrobials compared to AC-FCP or AC-FCP + antimicrobials fed birds. Serum albumin was lower (p<0.01) in AC-FCP birds than that in other birds. There was a tendency (p=0.09) that birds fed AC-FCP diet had lower total serum cholesterol than other birds.

**Conclusion:**

Feeding AC-FCP has potential to improve the intestinal health and protect the birds from acute infections.

## Introduction

Following the withdrawal of antimicrobial agents as feed additives from broiler diets, the broiler industry is now dealing with increasingly problems related to intestinal microbial imbalance and impaired immune competence [1,2]. Several nutraceutical compounds have been proposed to replace the role of synthetic antimicrobials for broiler chickens, of which probiotics and antioxidants are the examples [2]. Recently, we have isolated the filamentous fungus *Acremonium charticola* (AC) from the Indonesian fermented dried cassava [3] and showed that the fungus had probiotic [4] and antioxidant potentials [4,5]. The fungus may, therefore, be exploited to exert health effects on broiler chickens. Apart from its nutraceutical properties, AC has been reported to possess a fiber-degrading ability [4] that may be advantageous for lowering the fiber content of particular (unconventional) feed ingredients.

Due to global rise in the price of feed ingredients (for example maize), there is now a tendency in the poultry industry to move toward the use of alternative feed ingredients. Cassava pulp, which is a by-product of tapioca industry, has recently been used in broiler diets as an energy source. However, its utilization is limited due to the low and high contents of protein and fiber, respectively [6]. In the previous study, we fermented cassava pulp (FCP) with AC and subsequently included in broiler rations to partially replace maize in the diets. Indeed, feeding AC-FCP improved immune responses (indicated by the low heterophil to lymphocyte [H/L] and albumin to globulin [A/G] ratio) and had no negative effects on the nutritional state and metabolic performance of broiler chickens [7]. In this study, AC-FCP was provided to broilers not only to reduce the proportion of maize in the diets but also to replace the role of synthetic antimicrobials for broiler chickens.

Taken together, the objective of this study was to evaluate the effect of feeding AC-FCP on the intestinal microbial ecology and hematological indices of broiler chickens.

## Materials and Methods

### Ethical approval

The present study was approved by the Animal Ethics Committee of Faculty of Animal and Agricultural Sciences, Diponegoro University.

### Fungi and starter preparation

AC inoculum was prepared by retrieving the fungal culture (maintained on a potato-dextrose-agar [PDA; Merck KGaA, Darmstadt, Germany] and stored at 4°C), streaking on PDA medium and incubating at 38°C for 2 days. After being dislodged from the PDA, the fungal mycelia was diluted in 200 ml of sterilized distilled water and further used to prepare the fungal starter.

About 200 g of sterilized dry cassava pulp (87.5% dry matter) was inoculated with the suspension of fungal mycelia and then thoroughly mixed. After aerobic incubation at room temperature for 4 days, the inoculation starter was enumerated based on the colony counting method. The fungal starter produced was eventually used to ferment the cassava pulp for the *in vivo* experiment.

### FCP preparation

Fermentation of cassava pulp was carried out according to Sugiharto *et al*. [7] with some modifications. In brief, 10 kg of steamed cassava pulp was soaked with sterile water (1:1). The cassava pulp was inoculated with 110 g/kg fungal starter (3.6 × 10^10^ cfu/g) and 41 g/kg urea and then thoroughly mixed. The mixture was incubated for 4 days and turned every 2 days. The AC-FCP was sundried before use for the *in vivo* experiment. Proximate analysis showed that AC-FCP contained energy (based on Bolton [8] formula) of 2886 kcal/kg and crude protein (Kjeldahl method) 8.5%.

### *In vivo* experiment

A total of 240 male Lohman MB-202 day-old-chicks (body weight=41.3±2.68 g; mean±standard deviation) purchased from a local hatchery were placed in an open-sided naturally ventilated broiler house and randomly allotted to one of the four experimental diets, including control diet, control diet with antimicrobials (neomycin), diet containing AC-FCP or diet containing AC-FCP and antimicrobials. The diets were formulated to be isonitrogenous and isoenergy and meet the Indonesian National Standards for Broiler Feed [9] requirements for broilers (Table-1). The diets were fed *ad libitum* in mash form, and throughout the experimental period, the birds were reared in concrete floor pens with rice husk as bedding material equipped with round bottom feeder and manual drinker.

**Table-1:** Ingredients and composition (as-dry basis) of treatment diets.

Items (%, unless otherwise noted)	Dietary treatments			

	Control diet	Control diet+antimicrobials	AC-FCP	AC-FCP+antimicrobials
Maize	54.0	54.0	45.0	45.0
Soybean meal	27.0	27.0	23.5	23.5
AC-FCP	-	-	16.0	16.0
Fish meal	9.00	9.00	10.6	10.6
Rice bran	6.75	6.75	1.25	1.25
Broken rice	1.00	1.00	1.50	1.50
DL-methionine	0.23	0.23	0.25	0.25
L-lysine	0.06	0.06	0.15	0.15
Limestone	1.01	1.01	0.80	0.80
Dicalcium phosphate	0.20	0.20	0.20	0.20
Premi×1	0.50	0.50	0.50	0.50
NaCl	0.25	0.25	0.25	0.25
Neomycin	-	0.0003	-	0.0003
Calculated composition				
Metabolizable energy (kcal/kg)^2^	2892	2892	2873	2873
Crude protein	22.5	22.5	22.0	22.0
Crude fiber	3.52	3.52	5.67	5.67
Calcium	1.03	1.03	1.03	1.03
Phosphor	0.56	0.56	0.54	0.54
Methionine	0.66	0.66	0.66	0.66
Lysine	1.43	1.43	1.43	1.43

1 Mineral-vitamin premix provided (per kg of diet) Ca - 2.250 g, P - 0.625 g, Fe - 3.570 mg, Cu - 0.640 mg, Mn - 5.285 mg, Zn - 0.003 mg, Co - 0.001 mg, Se - 0.013 mg, I - 0.016 mg, vitamin A - 375 IU, vitamin D - 150 IU, vitamin E - 0.080 mg,

2 Values were obtained based on the formula according to Bolton [8]. AC-FCP=*Acremonium charticola*-fermented cassava pulp

At day 28, birds from each pen were randomly selected for sampling after being deprived from feed for 8 h. For hematological analysis, blood was obtained from the birds’ wing veins and collected in ethylenediamine tetraacetic acid-containing vacutainers. The rest of the blood was collected in vacutainers containing no anticoagulant and allowed to clot for 2 h at room temperature. After centrifugation at 2000 rpm for 15 min, the serum was obtained and stored at −20°C until serum biochemistry analysis. The same birds that were blood sampled were sacrificed after being weighed, and immediately the internal organs were removed and weighed. Digesta were aseptically obtained from the ileum and cecum for pH measurement and microbiological and short-chain fatty acids (SCFA) analyses.

Microbial counts in the intestinal digesta were determined as described by Engberg *et al*. [10] with few modifications. Total bacteria were counted on PDA after aerobic incubation at 38°C for 24 h. Coliform bacteria and lactose-negative enterobacteria were enumerated on MacConkey agar following aerobic incubation at 38°C for 24 h as red and colorless colonies, respectively. Enterobacteria were the number of coliform bacteria and lactose-negative enterobacteria. Lactic acid bacteria (LAB) were enumerated on de Man, Rogosa and Sharpe agar after anaerobic incubation at 38°C for 48 h. The concentration of SCFAs in the cecal contents was determined by gas chromatography under the conditions described by Sugiharto *et al*. [11].

The numbers of erythrocytes and leukocytes were measured based on the dilution flask method, and a Bürker chamber was employed to count corpuscles. The concentration of hemoglobin was determined by Sahli’s method, and hematocrit values were estimated through the microhematocrit technique. The differential leukocytes of broilers were determined using a light microscope with an immersion lens. Coverslip technique was performed when preparing blood smears. H/L ratio was determined by dividing the numbers of H/L. Serum total triglyceride was determined according to enzymatic colorimetric method using glycerol-3-phosphateoxidase (DiaSys Diagnostic System GmbH, Holzheim, Germany). Total cholesterol, high-density lipoprotein (HDL), and low-density lipoprotein (LDL) cholesterol were measured based on enzymatic colorimetric method with cholesterol oxidase/p-aminophenazone (DiaSys Diagnostic System GmbH, Holzheim, Germany). The enzymes of alanine aminotransferase (ALT) and aspartate aminotransferase (AST) were determined spectrophotometrically using a Reflotron system (Roche Diagnostics Corporation, Indianapolis, IN, USA). Total protein in the serum was assessed by photometric test based on the biuret method with the kit (total protein kit, DiaSys Diagnostic System GmbH, Holzheim, Germany) according to the manufacturer’s instructions. Albumin in serum was measured by photometric test using bromocresol green (DiaSys Diagnostic System GmbH, Holzheim, Germany). Globulin was obtained by subtracting albumin values from total serum protein. A/G ratio was calculated by dividing albumin and globulin values.

### Data analysis

The data were analyzed according to a completely randomized design by ANOVA using the General Linear Models Procedure in SAS (SAS Inst. Inc., Cary, NC, USA). Pen was treated as the experimental unit. Results are presented as least square means and standard error of the mean. Significant differences among dietary treatments were further analyzed with Duncan’s multiple-range test. A significant level of p≤0.05 was applied.

## Results

### Intestinal microbial population

The data of microbial population in the ileal and cecal digesta of broilers are presented in Table-2. Birds fed AC-FCP had lower (p<0.05) coliform bacteria count in the ileal digesta when compared with the birds fed control diet or control diet + antimicrobials, but the significant difference was not observed when compared with those of fed AC-FCP + antimicrobials. The difference in coliform bacteria was not seen in the cecal digesta of broilers. There was no difference (p>0.05) with regard to total bacteria, enterobacteria, lactose negative-enterobacteria, and LAB in the ileal and cecal digesta of broilers across the treatment groups.

**Table-2:** Microbial population in the ileal and cecal digesta of broilers fed the treatment diets.

Items (log cfu/g)	Dietary treatments				SEM	p value

	Control diet	Control diet+antimicrobials	AC-FCP	AC-FCP+antimicrobials		
Ileum						
Total bacteria	12.1	12.2	12.2	12.2	0.19	0.94
Enterobacteria	6.71	6.67	6.35	6.39	0.17	0.35
Coliform bacteria	6.61^a^	6.61^a^	5.95^b^	6.28^ab^	0.16	0.03
Lactose negative-enterobacteria	5.95	5.82	5.97	5.63	0.24	0.74
LAB	8.44	8.60	8.28	8.29	0.13	0.27
Cecum						
Total bacteria	12.1	12.3	12.1	11.9	0.18	0.54
Enterobacteria	6.53	6.46	6.55	6.41	0.08	0.61
Coliform bacteria	6.40	6.33	6.38	6.25	0.08	0.56
Lactose negative-enterobacteria	5.88	5.76	5.97	5.86	0.16	0.82
LAB	8.46	8.46	8.44	8.29	0.12	0.69

^a, b^ Means in a row with different superscripts are significantly different (p≤0.05). AC-FCP=*Acremonium* *charticola*-fermented cassava pulp, LAB=Lactic acid bacteria, SEM=Standard error of mean

### Concentrations of SCFAs in ceca

The concentrations of SCFA in digesta from the ceca of broilers are presented in Table-3. Butiric acid was higher (p<0.05) in the cecal digesta of birds fed AC-FCP than in the digesta from other birds. Accordingly, the concentration of propionic acid was higher in the digesta of AC-FCP birds than in other birds although it was not statistically significant. The significant difference was not observed for the concentration of acetic acid and the pH of digesta from ceca of broilers.

**Table-3:** Concentrations of SCFA and pH of cecal digesta of broilers fed the treatment diets.

Items	Dietary treatments				SEM	p value

	Control diet	Control diet+antimicrobials	AC-FCP	AC-FCP+antimicrobials		
Acetic acid (mmol/kg)	27.4	44.6	37.2	47.5	9.07	0.34
Propionic acid (mmol/kg)	10.6	10.3	19.0	13.4	2.81	0.12
Butiric acid (mmol/kg)	4.39a	6.66^ab^	10.5^b^	4.77^a^	1.45	0.03
pH of ceca	6.00	5.40	5.80	6.00	0.32	0.51

^a, b^ Means in a row with different superscripts are significantly different (p≤0.05). AC-FCP=*Acremonium charticola*-fermented cassava pulp, SEM=Standard error of mean, SCFA=Short chain fatty acids

### Hematological and biochemical parameters

The data of hematological and biochemical parameters of broilers are presented in Table-4. The percentages of lymphocytes and heterophils were higher (p<0.05) and tended (p=0.07) to be lower, respectively, in broilers fed control diet than in other birds. The birds provided control diet had lower (p<0.05) H/L ratio as compared with those of fed AC-FCP or AC-FCP + antimicrobials. There was no difference (p>0.05) in the numbers of hemoglobin, erythrocytes, hematocrit, mean corpuscular volume, mean corpuscular hemoglobin (MCH), MCH concentration, and leukocytes as well as the proportion of eosinophils and monocytes in the blood of broiler chickens.

**Table-4:** Hematological and biochemical parameters of broilers fed the treatment diets.

Items	Dietary treatments				SEM	p value

	Control diet	Control diet+antimicrobials	AC-FCP	AC-FCP+antimicrobials		
Hematological parameters						
Hemoglobin (g/dL)	6.92	6.65	7.40	7.10	0.54	0.77
Erythrocytes (10^12^/L)	2.67	2.50	2.82	2.49	0.21	0.57
Hematocrit (%)	20.2	19.5	22.0	20.8	1.68	0.72
MCV (fl)	76.6	78.7	80.0	85.7	8.82	0.86
MCH (pg)	26.2	26.8	26.9	29.3	2.94	0.86
MCHC (g/dL)	34.4	34.1	33.7	34.1	0.24	0.16
Leukocytes (10^9^/L)	16.4	17.4	10.9	14.5	2.63	0.29
Heterophils (%)	17.4	22.3	29.4	26.3	3.83	0.07
Eosinophils (%)	1.40	3.75	2.20	4.25	0.88	0.10
Lymphocytes (%)	73.6^a^	57.3^b^	54.6^b^	55.4^b^	5.59	0.05
Monocytes (%)	7.60	8.25	13.8	9.0	2.19	0.15
H/L ratio	0.24^a^	0.35^ab^	0.54^b^	0.50^b^	0.09	0.05
Serum biochemical parameters						
AST (U/L)	280	239	249	207	22.8	0.20
ALT (U/L)	13.6	16.9	13.2	15.5	1.43	0.24
Total triglyceride (g/dL)	47.2	43.6	39.2	47.4	6.72	0.80
Total cholesterol (g/dL)	137	130	103	111	10.0	0.09
LDL (g/dL)	21.2	11.5	12.5	7.85	5.92	0.46
HDL (g/dL)	100	111	89.3	96.4	9.87	0.17
Total protein (g/dL)	3.08^a^	3.12^a^	2.14^c^	2.57^b^	0.12	<0.01
Albumin (g/dL)	0.94^a^	0.89^a^	0.59^b^	0.81^a^	0.06	<0.01
Globulin (g/dL)	2.15^a^	2.23^a^	1.55^b^	1.76^b^	0.09	<0.01
A/G ratio	0.45	0.41	0.38	0.46	0.03	0.13

^a, b^ Means in a row with different superscripts are significantly different (p≤0.05). AC-FCP=*Acremonium* *charticola*-fermented cassava pulp, MCV=Mean corpuscular volume, MCH=Mean corpuscular hemoglobin, MCHC=Mean corpuscular hemoglobin concentration, H/L ratio=Heterophils to lymphocytes ratio, AST=Aspartate transaminase, ALT=Alanine aminotransferase, LDL=Low-density lipoprotein, HDL=High-density lipoprotein, A/G ratio=Albumin to globulin ratio, SEM=Standard error of mean

Serum total protein and globulin were higher (p<0.01) in birds fed control diet or control diet + antimicrobials as compared to those of fed AC-FCP or AC-FCP + antimicrobials. Serum albumin was lower (p<0.01) in AC-FCP birds than that in other birds. There was a tendency (p=0.09) that birds fed AC-FCP diet had lower total cholesterol in the serum than other birds. No significant different was observed for the serum AST, ALT, total triglyceride, LDL, HDL and A/G ratio of broilers.

### Internal organ weight

The data of internal organ weight of broilers are presented in Table-5. In general, no significant difference of internal organ weight of broilers was observed in this study.

**Table-5:** Internal organ weight of broilers fed the treatment diets.

Items (% body weight)	Dietary treatments				SEM	p value

	Control diet	Control diet+antimicrobials	AC-FCP	AC-FCP+antimicrobials		
Spleen	0.12	0.20	0.16	0.17	0.05	0.68
Thymus	0.21	0.28	0.25	0.26	0.03	0.40
Bursa of fabricius	0.23	0.25	0.28	0.27	0.04	0.72
Liver	2.93	2.97	2.74	2.65	0.16	0.43
Giblets^1^	6.57	6.82	5.95	5.94	0.32	0.12
Duodenum	1.22	1.30	1.34	0.86	0.16	0.18
Jejunum	2.09	2.06	1.74	1.85	0.23	0.67
Ileum	1.46	1.79	1.42	1.40	0.16	0.28
Ceca	0.61	0.53	0.40	0.65	0.09	0.23

1 Giblets: Heart, gizzard and liver, AC-FCP=*Acremonium charticola*-fermented cassava pulp, BW=Body weight, SEM=Standard error of mean

## Discussion

Fermentation of cassava pulp with the fungus AC was expected to produce a cheap functional feed that may be used to replace the role of synthetic antimicrobials for broiler chickens. Our present results showed that feeding AC-FCP reduced the number of coliform bacteria in the ileal digesta of broilers. This finding was in accordance with our previous study reporting the potential of AC in inhibiting the growth of *Escherichia coli* *in vitro* [4]. The mechanism through which AC reduced the population of coliform bacteria in the ileum of broilers remains unclear, but the capability of the fungus to produce some form of antibiotics and antimicrobial compounds that may impair the biological functions of the bacteria could be one mechanism [12]. Recent study reported that antioxidants are useful to modulate the cecal microflora and improve the immune competence of broiler chickens [13]. AC has been reported to possess antioxidant properties corresponding to ascorbic acid [5]. Hence, it is safe to assume that antioxidant activity of AC may also take part in lowering the number of coliform bacteria in the ileal contents of broilers. With regard to neomycin, this aminoglycoside antibiotic has been used to control the outbreak of *E. coli* infections in broilers [14]. However, our result showed no effect of neomycin on the population of coliform bacteria in the intestinal contents. The exact cause of this condition was not fully known, but the preparation of feed in mash form perhaps caused some of neomycin was not consumed by the chickens. As a consequence, the dose of neomycin was not enough to inhibit the proliferation of coliform bacteria in the intestine.

Volatile fatty acids (VFA) have been attributed to the health status of the gastrointestinal tract of chickens [15,16]. Of the VFA produced in cecum, butiric acid is of particular importance due to its nutritional properties for the intestinal epithelial cell. Butyric acid also has pathogen inhibitory effects in the intestine of broilers [16]. In addition to butyric acid, propionic acid has been reported to play an important role in inhibiting the colonization of birds by pathogenic bacteria [15,16]. In this study, birds fed AC-FCP produced higher butiric and propionic acids than other birds. It has been reported that the cecal concentration of VFA can be affected by several factors and that probiotics [17,18], prebiotics and synbiotics [17] may elevate the concentration of VFA in the ceca of broilers. Taken together, probiotic activity of AC growing in the FCP seemed to be responsible for the increase in concentrations of butyric and propionic acids in the cecum of broilers. To date, the mechanism by which AC increased the concentration of butyric and propionic acids is not fully understood, but Meimandipour *et al*. [16] suggested that probiotic microorganisms may elevate the population of anaerobic bacteria responsible for producing butyric and propionic acids in the cecum of broilers. The increased concentration of butyric acid was, however, not seen in birds that received diet containing AC-FCP + antimicrobials. In such case, neomycin may interrupt the balance of cecal microflora [19] resulting in reduced butyrate production. However, this inference should be interpreted with caution as most aminoglycoside antibiotics including neomycin are mostly ineffective against anaerobic bacteria such as butyric acid-producing bacteria in the cecum.

In this study, the proportion of lymphocytes was higher in broilers fed the control diet (without antimicrobials) than in birds fed AC-FCP or diets with antimicrobials. Indeed, the percentage of lymphocytes in control birds was higher than the standard of lymphocytes in chickens (63%, [20]). This condition may indicate that control birds experienced infection, as Ogunleye *et al*. [21] suggested that the increased numbers of lymphocytes (lymphocytosis) can be an indicator of acute infection and internal stress in chickens. Concomitant with the latter authors, Dudek and Bednarek [22] reported that the consecutive injection with *E. coli* lipopolysaccharides (LPS) resulted in lymphocytosis in pigeons. In this study, the concentration of globulin was higher in the serum of control birds when compared especially with the birds fed AC-FCP. Sharma *et al*. [23] previously reported an increased globulin concentration in the group of broilers infected with *E. coli* O78 as compared with uninfected broilers. The increased globulin in the present study may therefore further support our inference that control birds suffered from infection. The proportion of heterophils tended to be lower in control birds than in birds receiving AC-FCP or diets with antimicrobials in the current study. The heterophils in control birds were by far lower from the standard of heterophils in chickens (30.1%, [20]). It has been reported in pigeons that infection (consecutive injection with *E. coli* LPS) resulted in decreased number of heterophils (heteropenia) [22]. The latter authors further suggested that acute infection may increase migration of most heterophils from circulation into tissues resulting in decreased proportion of heterophils in the blood. H/L ratio has long been used to indicate mild to moderate stress in poultry [7,24]. In this study, H/L ratio was lower in birds fed control diet as compared with other birds. However, it is difficult to infer that control birds had lower level of stress than other birds as Müller *et al*. [25] suggested that severe stress (e.g., due to acute infections) may cause heteropenia and lymphocytosis in the periphery resulting in low H/L ratio. Overall, from the microbiological and hematological data, it can be suggested that feeding AC-FCP may be beneficial for broilers in preventing the infection by pathogens. This suggestion was supported by Engberg *et al*. [26] at which fermented feed protected the chickens from *E. coli*, *Salmonella* and *Campylobacter* infections.

It has been reported that probiotics can lower total cholesterol level in the serum of broiler chickens [27]. In accordance with this, there was a clear tendency in the present study that birds receiving AC-FCP had lower total cholesterol level in the serum compared with other birds. Apart from the benefit effects of AC-FCP to broilers as mentioned above, feeding such diet should be conducted with caution as it could decrease total protein and albumin concentrations in the blood of broilers. In the preparation of AC-FCP, urea was used to increase the crude protein content of AC-FCP. Perhaps, some of the crude protein derived from urea could not be utilized properly by the chickens resulting in lower level of total protein and albumin in the serum of AC-FCP fed birds [28]. However, the decreased level of blood protein and albumin did not cause adverse effect on the internal organ development in this study.

## Conclusions

It can be concluded from the present study that inclusion of AC-FCP in broiler diets was potential to improve the intestinal health and protect the birds from acute infections. Hence, AC-FCP can be a functional feed that may be used to replace the role of synthetic antimicrobials for broiler chickens.

## Authors’ Contributions

SS designed the study, analyzed the results, drafted and revised the manuscript. TY, II, EW and FDP carried the animal experiment and performed the laboratory work. All authors read and approved the final manuscript.
